# 17β-Estradiol Stimulates Oxidative Stress Components and Thyroid Specific Genes in Porcine Thyroid Follicular Cells: Potential Differences Between Sexes

**DOI:** 10.3390/cells13211769

**Published:** 2024-10-25

**Authors:** Jan Stępniak, Małgorzata Karbownik-Lewińska

**Affiliations:** 1 Department of Endocrinology and Metabolic Diseases, Medical University of Lodz, 93-338 Lodz, Poland; jan.stepniak@umed.lodz.pl; 2 Polish Mother’s Memorial Hospital-Research Institute, 93-338 Lodz, Poland

**Keywords:** thyroid follicular cell, 17β-estradiol, sexual dimorphism, thyroperoxidase, NOX, DUOX, endoplasmic reticulum, reactive oxygen species

## Abstract

17β-estradiol plays a crucial role in regulating cellular processes in both reproductive and non-reproductive tissues, including the thyroid gland. It modulates oxidative stress and contributes to sexual dimorphism in thyroid diseases, with ROS production, particularly H_2_O_2_, generated by NOX/DUOX enzymes. This study aimed to investigate the effects of 17β-estradiol (10 nM or 100 nM) on the expression of NOX/DUOX, thyroid-specific genes, and endoplasmic reticulum (ER) stress-related genes in male and female porcine thyroid follicular cells. Expression of the studied genes was evaluated by RT-PCR before and after treatment with 17β-estradiol alone or with the addition of NOX4 inhibitor (GKT-136901). Additionally, the level of ROS was measured by flow cytometry analysis. Our results show that 17β-estradiol significantly upregulates thyroid-specific genes, particularly TPO, and stimulates NOX/DUOX expression, affecting the redox state of thyroid cells. It also stimulates ER stress-related genes such as CHOP. In conclusion, estrogen excess may contribute to thyroid disease development via such possible mechanisms as the upregulation of key thyroid-specific genes, particularly TPO, and of genes involved in the cellular response to ER stress, especially CHOP, as well as by the stimulation of the NOX/DUOX system with consequent ROS overproduction. These mechanisms may play a certain role in the higher prevalence of thyroid diseases in women.

## 1. Introduction

Estrogens, steroid hormones derived from cholesterol, play crucial roles in both the reproductive and non-reproductive systems of females and males [1]. The most biologically active and prevalent estrogen is 17β-estradiol, produced predominantly in the ovary and, to a lesser extent, in the testes, adrenals, and adipose tissue [1]. By binding to estrogen receptors found in various tissues, 17β-estradiol initiates molecular events that regulate gene expression and cellular function, mediating key cellular processes such as proliferation, differentiation, motility, and apoptosis. This influence extends beyond reproductive tissues such as the breast, endometrium, ovary, and testes to non-reproductive tissues, including the endocrine glands, where 17β-estradiol regulates metabolic processes, cardiovascular health, bone density, and brain function [2].

Moreover, 17β-estradiol significantly impacts the cellular redox state, demonstrating both pro- and antioxidative properties. This ability to modulate oxidative stress is crucial in preventing cellular damage implicated in diseases such as cardiovascular disorders [3] and neurodegenerative conditions [4]. Under experimental in vitro conditions, estrogens protect against reactive oxygen species (ROS)-induced damage in the reproductive [5,6] and non-reproductive [7] tissues. They contribute to cellular health and longevity by promoting vascular protection through nitric oxide production [8]. On the other hand, results of epidemiological and experimental research indicate that 17β-estradiol can play a role in cancer development in estrogen-responsive tissues such as the breast and endometrium by promoting cell proliferation, inhibiting apoptosis, and inducing DNA damage through oxidative stress [9].

Also in the thyroid gland, 17β-estradiol has been shown to be a potent stimulator of both benign and malignant human thyroid cells, significantly influencing their growth and function [10,11]. This role as a growth factor may help explain why proliferative thyroid diseases are more prevalent in females than in males. Both benign and malignant thyroid tumors are 3–4 times more likely to develop in women than in men, and thyroid autoimmune diseases also have a higher prevalence in women [12,13,14]. Given that 17β-estradiol can exert its effects through multiple pathways, determining the precise mechanisms by which it impacts the thyroid is challenging and the exact causes and mechanisms underlying this sexual dimorphism remain unknown. Recent experimental studies suggest that ROS, such as hydrogen peroxide (H_2_O_2_), produced as a result of 17β-estradiol action, may play a critical role in the sexual dimorphism observed in thyroid diseases [15,16]. It is hypothesized that oxidative stress could be a key factor underlying both proliferative and autoimmune thyroid conditions [17,18].

H_2_O_2_ is primarily a toxic compound, and its excess can lead to DNA oxidation, resulting in oxidative damage, mutagenesis, and apoptosis. Studies have shown that the thyroid gland experiences higher levels of DNA oxidative damage and spontaneous mutations compared to other tissues, potentially due to prolonged exposure to H_2_O_2_ [19].

In thyroid follicular cells (thyrocytes) the major non-mitochondrial sources of H_2_O_2_ are transmembrane proteins belonging to the NADPH oxidase/dual oxidase (NOX/DUOX) family, including DUOX1, DUOX2, and NOX4. DUOX1 and DUOX2, localized at the apical membrane of thyrocytes, generate H_2_O_2_ necessary for thyroid hormone biosynthesis, with their activity dependent on intracellular calcium levels. NOX4, in contrast, is active across various cellular compartments, including the endoplasmic reticulum (ER), and its ROS-generating activity is constitutive, depending only on its expression levels. The physiological function of H_2_O_2_ produced by NOX4 in the normal thyroid gland is not fully understood; however, studies indicate that NOX4 acts as a modulator of differentiation in thyroid normal cells as its expression is inversely correlated with thyroid-specific genes such as sodium/iodide symporter (NIS), thyroperoxidase (TPO), or paired box transcription factor 8 (PAX8) [20]. NOX4 in the thyroid gland may also play a key role in the functioning of the ER, which is essential for the primary function of thyrocytes, i.e., the secretion of thyroid hormones. The ER is responsible for the synthesis, folding, and quality control of secretory proteins, and disruptions in this process can lead to so-called ER stress, negatively affecting normal cellular functions. To manage this stress, cells activate the unfolded protein response (UPR), i.e., an intracellular signaling pathway that helps maintain ER homeostasis. It has been shown that, in endothelial cells, NOX4 significantly contributes to the production of ER-localized H_2_O_2_, which serves as a signal to initiate the UPR and autophagy [21]. These processes may be particularly important in thyrocytes, as ER stress has been linked to the development of autoimmune diseases, which was documented in numerous studies [22].

Research conducted by our team, among others, has highlighted the significant role of 17β-estradiol in oxidative processes within the thyroid gland [16]. In a study using primary cell cultures derived from the thyroid glands of adult male and female Wistar rats, we observed that the expression of DUOX1 and NOX4 was significantly higher in cells from female thyroids compared to those from male glands [16]. These findings align with previous experimental studies showing sexual dimorphism in the expression of NOX4 [15], suggesting that estrogens may regulate the expression of these genes. Additionally, NOX4 has been found to be overexpressed in thyroid cancer, linking this H_2_O_2_-generating system to cancer pathogenesis [23].

The aim of the present study was to investigate the effects of 17β-estradiol on the expression of thyroid NOX/DUOX, thyroid-specific genes, and genes involved in ER stress and UPR initiation in primary thyroid follicular cells from male and female porcine thyroids. Additionally, the study aimed to evaluate the contribution of NOX4, and the H_2_O_2_ it produces, in modulating thyroid-specific genes and those related to ER stress and UPR initiation, thereby assessing the impact of NOX4 on thyroid physiology. Furthermore, the effects of exogenous 17β-estradiol between sexes were compared.

Unlike in our previous studies, we changed the research model due to the large number of rat thyroids (and thus euthanized rats) required, which posed both economic and ethical concerns. Additionally, redox gene expression in rats only partially corresponds to that in humans, limiting the clinical relevance of the rat thyroid cell model [24]. Pigs, however, are an ideal animal model for human health research because their anatomy and physiology closely resemble those of humans. Specifically, the porcine thyroid shares similar weight, dimensions, and structure with the human thyroid. Moreover, the porcine genome is three times closer to the human genome than that of rodents, providing a more relevant genetic model [25]. Given these advantages and the challenge of obtaining sufficient healthy human thyroid tissue for primary cell culture, we opted to use primary cell lines derived from porcine thyroid glands.

## 2. Materials and Methods

### 2.1. Materials

Most reagents and materials were purchased from the same company (Sigma-Aldrich, Saint Louis, MO, USA); other cases are specified.

### 2.2. Animals

We collected the porcine thyroids glands from three adult male and three adult female animals at a slaughterhouse. All procedures associated with the killing of the animals were in agreement with the European Community Council Regulation (CE1099/2009). It should be stated that it is not required to obtain any approval from the Local Ethics Committee when tissues and organs are collected from animals that are only subject to registration by the center in which the organs or tissues were taken, in agreement with Directive 2010/63/EU of the European Parliament and the Council on the protection of animals used for scientific purposes (published on 22 September 2010) and also with the Polish Act on the Protection of Animals Used for Scientific or Educational Purposes (published on 15 January 2015). The average age of the animals was 8–9 months, which means that they were sexually mature. Their body mass was 125.35 ± 4.8 (SD) kg. The sex of the animals was determined by a veterinarian based on primary sexual characteristics, such as the position of the urogenital opening. This determination was further confirmed after chest opening and evisceration by the presence or absence of the uterus and ovaries. According to the veterinarian responsible for animal health and slaughterhouse hygiene, all animals were in good overall condition and showed no signs of pathological issues. The thyroid glands were collected after the slaughter (within 20 to 25 min), placed in Hanks’ balanced salt solution (HBSS), and kept in such a way until the next procedure.

### 2.3. Cell Culture

We removed any adherent connective tissue from thyroid tissue. Then, the examined tissue was placed in a cell culture hood and minced (with the application of a sterile razor blade) to obtain smaller fragments of approximately 1–3 mm. After two washes in HBSS, thyroid fragments were digested in collagenase IV (2 mg/mL; 1.5–3.0 h, 37 °C). Thereafter, to remove undigested tissue fragments, the obtained material was filtered through a nylon mesh (100 μm). After two washes in HBSS and then centrifugation (1000× *g*, 5 min), the cells were seeded to a density of 1 × 10^6^ cells in 1 mL of culture medium per well in 6-well plates. The culture medium consisted of DMEM + GlutaMAX without Pyruvate (Dulbecco’s modified Eagle’s medium; Gibco) supplemented with 10% fetal bovine serum (FBS, Gibco), thyroid-stimulating hormone (TSH; 1 mIU/mL), penicillin-streptomycin (100 IU/mL), and amphotericin B (2.5 µg/mL). After 72 h of incubation (37 °C, air:CO_2_ (95:5%) atmosphere), the examined substance/substances were added.

### 2.4. Cell Treatment

Twenty-four hours before the experiments, the medium was replaced with a FluoroBrite™ DMEM medium supplemented with FBS. The cells of male or female origin were treated with 17β-estradiol in concentrations of 0.0 nM, 10 nM, or 100 nM, with or without the addition of the NOX4 inhibitor (GKT-136901; Sigma-Aldrich, Saint Louis, MO, USA) in the concentration of 20 µM. Stock solutions of 17β-estradiol (10 µM and 100 µM) and of GKT-136901 were prepared in dimethyl sulfoxide (DMSO). Thus, control cells were also treated with DMSO (in the final concentration of 0.1%). The cells were incubated for another 48 h in the presence of examined substances.

### 2.5. Cell Viability Assay

A cell viability test was conducted using the eBioscience™ Annexin V Apoptosis Detection Kit (Invitrogen, Waltham, MA, USA) according to the manufacturer’s instructions and analyzed via flow cytometry with a FACSCanto II cytometer (BD FACSCanto II, San Diego, CA, USA). The data were processed using FACSDiva software 6.1.2 (BD, San Diego, CA, USA).

### 2.6. Evaluation of ROS Level

The level of intracellular ROS was measured with the use of CellROX™ Orange Reagent (ThermoFisher, Waltham, MA, USA) according to the manufacturer’s protocols. It is a novel fluorogenic probe for measuring oxidative stress in live cells. The cell-permeant dye is weakly fluorescent while in a reduced state and exhibits bright orange photostable fluorescence upon oxidation by ROS. Briefly, cells were treated with CellROX™ Orange Reagent at a final concentration of 5 µM and incubated for 30 min at 37 °C. After incubation, cells were dissociated with the use of TrypLE™ (Gibco) enzyme and subjected to flow cytometry. Flow cytometry analysis was performed using the FACSCanto II cytometer (BD FACSCanto II, San Diego, CA, USA). Analysis of data was performed using the FACSDiva software (BD, San Diego, CA, USA).

### 2.7. mRNA Analysis by qRT-PCR

This step was described in detail in [16]. The differences are as follows. RNA concentration and purity were measured using the NanoDrop™ 2000 Spectrophotometer (ThermoFisher Scientific). Real-time PCR was performed on the 7900HT Fast Real-Time PCR System (Applied Biosystems, Waltham, MA, USA). The Assay Identification numbers were: NOX4—Ss06909549_m1; DUOX1—Ss03394039_m1; DUOX2—Ss03393368_m1; ER degradation enhancing alpha-mannosidase-like protein 1 (EDEM)—Ss06906535_m1; Heat shock protein family A (Hsp70) member 5 (BIP, GRP78)—Ss03374255_m1; DNA damage inducible transcript 3 (CHOP)—Ss03821509_s1; Activating transcription factor 4 (atf4)—Ss03390207_g1; DNA heat shock protein family (Hsp40) member C3 (P58IPK)—Ss00939342_m1; NIS, solute carrier family 5 member 5 (SLC5A5)—Ss03394915_m1; Thyroid peroxidase (TPO)—Ss03374684_u1; Thyroglobulin (Tg)—Ss04245888_m1 and NK2 homeobox 1 (NKX2-1) –Ss04954747_m1. Ywhaz—Ss03216374_g1 was used as the endogenous control. The expression levels of the genes under investigation in treated cells were quantified in comparison to their expression in untreated cells or male cells. The fold change in gene expression, normalized to an endogenous control, was calculated using the formula: RQ = 2^−ΔΔCT^. The results are presented as the log2 of the fold change (RQ) values.

### 2.8. Statistical Analysis

We used either the unpaired *t*-test or the one-way analysis of variance test followed by the Newman-Keuls multiple comparison test to determine statistically significant differences. The results of the qRT-PCR are presented as a log2 of fold changes (RQ) value relative to either the control cells (untreated) or to male cells. The results are presented as mean ± S.E. of three independent experiments. Statistically significant differences are accepted at the level of *p* < 0.05.

## 3. Results

### 3.1. Stimulation of Thyroid NADPH Oxidases Expression in Male/Female Thyroid Cells

**Figure 1:**
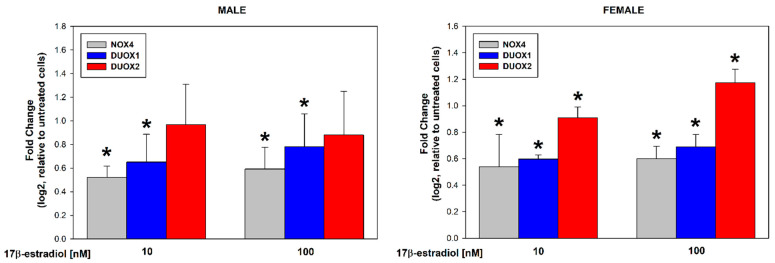
log2 fold change representing the mRNA expression of NOX4, DUOX1, and DUOX2 in cells derived from male or female porcine thyroid. Cells were incubated with 17β-estradiol at concentrations of 0.0 nM, 10 nM, or 100 nM. mRNA expression was calculated relative to the untreated control cells. Bars represent the mean ± SE of three independent experiments. * *p* < 0.05 vs. respective control, i.e., without 17β-estradiol (fold change equal to “0”).

**Figure 2:**
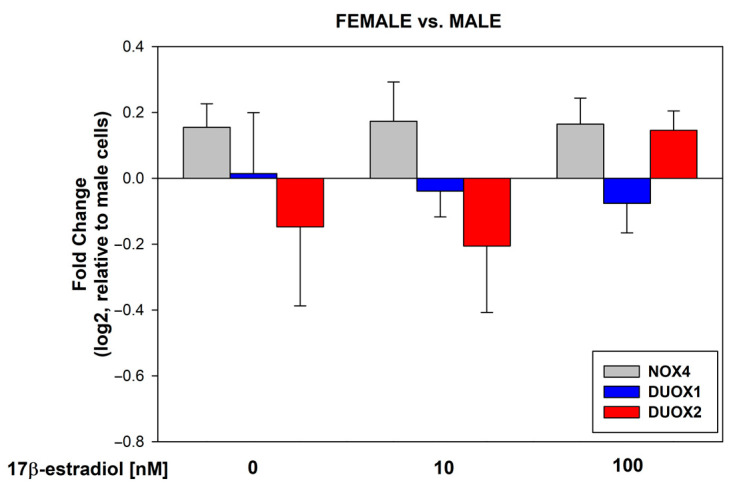
log2 fold change representing the mRNA expression of NOX4, DUOX1, and DUOX2 in female porcine thyroid cells relative to male porcine thyroid cells. Cells were incubated with 17β-estradiol at concentrations of 0.0 nM, 10 nM, or 100 nM. Bars indicate the mean ± SE of three independent experiments.

17β-estradiol concentrations of 10 and 100 nmol/L were added to the male and female thyroid primary cell lines for 48 h to assess its effect on the expression of NOX4, DUOX1, and DUOX2. The analysis of the RT-PCR results showed that 17β-estradiol causes a dose-dependent increase in the expression of NOX4 and DUOX1 in cells from both male and female thyroids. Expression of DUOX2 was significantly increased only in female thyroid cells (Figure 1). We found that 17β-estradiol was equally potent in its stimulating effects in both sexes (Figure 2). We have not observed statistically significant differences in the expression of NOX4, DUOX1, and DUOX2 between male and female thyroid cells (Figure 2).

### 3.2. Stimulation of ROS Formation by 17β-Estradiol in Male/Female Thyroid Cells

**Figure 3:**
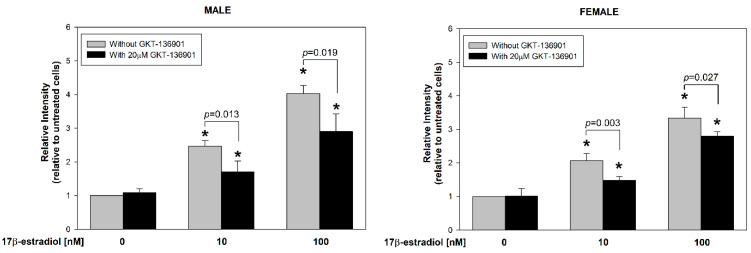
The level of ROS in cells derived from male or female porcine thyroid. Cells were incubated after the addition of 17β-estradiol (0.0 nM, 10 nM, or 100 nM) with or without GKT-136901 (20 µM). The level of ROS was evaluated by flow cytometry analysis with the use of CellROX™ Orange Reagent and was expressed relative to the untreated control group. Bars represent the mean ± SE of three independent experiments. * *p* < 0.05 vs. respective control (without 17β-estradiol).

**Figure 4:**
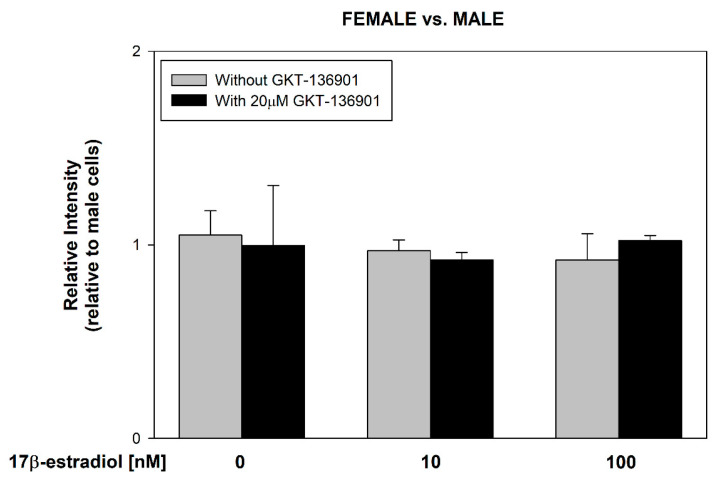
The level of ROS in cells derived from male and female porcine thyroid. Cells were incubated after the addition of 17β-estradiol (0.0 nM, 10 nM, or 100 nM) with or without GKT-136901 (20 µM). The level of ROS was evaluated by flow cytometry analysis with the use of CellROX™ Orange Reagent and was expressed relative to the respective male groups.

17β-estradiol concentrations of 10 and 100 nmol/L were added to the male and female thyroid primary cell lines for 48 h to assess its effect on ROS formation. Additionally, to evaluate the involvement of NOX4 in this process, GKT-136901 (selective NOX4 inhibitor) was used in the concentration of 20 µM. The level of intracellular ROS was measured by flow cytometry analysis with the use of CellROX™ Orange Reagent. We found that 17β-estradiol causes a dose-dependent increase in ROS formation (Figure 3). Moreover, GKT-136901 caused a reduction in ROS levels, both in male and female cells incubated with 10 and 100 nM of 17β-estradiol, respectively. Additionally, we have not found differences in ROS formation caused by 17β-estradiol between sexes (Figure 4).

### 3.3. Stimulation of Thyroid Specific Genes Expression by 17β-Estradiol in Male/Female Thyroid Cells

**Figure 5:**
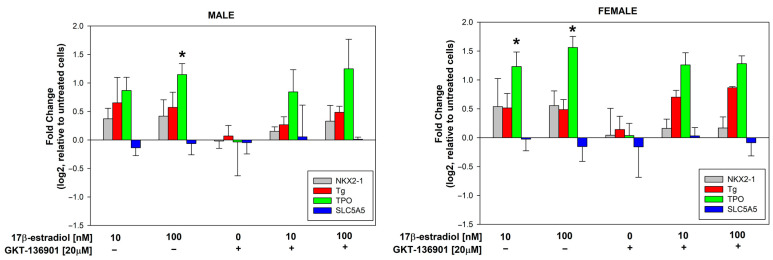
log2 fold change signifying the mRNA expression of thyroid-specific genes in cells derived from male and female porcine thyroid. Cells were incubated with 17β-estradiol at concentrations of 0.0 nM, 10 nM, or 100 nM, with or without GKT-136901 (20 µM). mRNA expression was calculated relative to the untreated control cells. Bars represent the mean ± SE of three independent experiments. * *p* < 0.05 vs. respective control (without 17β-estradiol).

**Figure 6:**
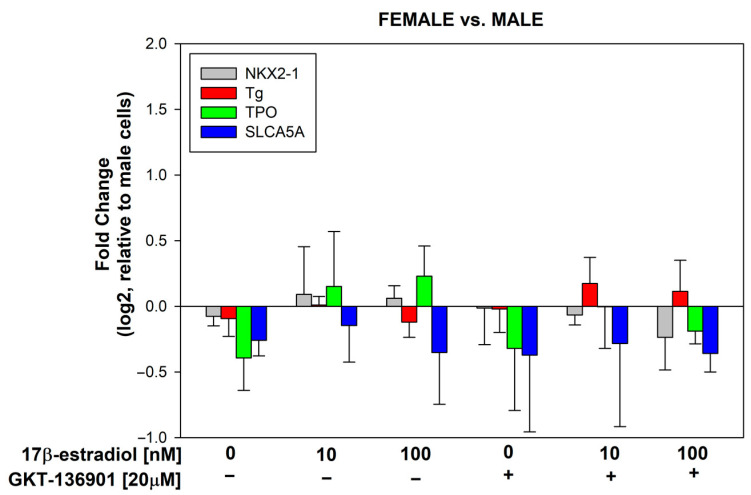
log2 fold change representing the mRNA expression of thyroid-specific genes in female porcine thyroid cells relative to male porcine thyroid cells. Cells were incubated with 17β-estradiol at concentrations of 0.0 nM, 10 nM, or 100 nM, with or without GKT-136901 (20 µM). Bars represent the mean ± SE of three independent experiments.

17β-estradiol concentrations of 10 and 100 nmol/L were added to the male and female porcine thyroid primary cell lines for 48 h to assess its effect on the expression of thyroid-specific genes, i.e., NKX2-1, Tg, TPO, and SLC5A5 (NIS). Additionally, to evaluate the potential involvement of NOX4 in the processes of thyroid stimulation, GKT-136901 (selective NOX4 inhibitor) was used in the concentration of 20 µM. The analysis of the RT-PCR results showed that 17β-estradiol in a concentration of 100 nM causes an increase in the expression of the TPO gene in cells from both male and female thyroids (Figure 5). The use of GKT-136901 did not change the level of thyroid-specific gene expression (Figure 6).

### 3.4. Stimulation of ER Stress/UPR Initiation Markers Expression by 17β-Estradiol in Male/Female Thyroid Cells

**Figure 7:**
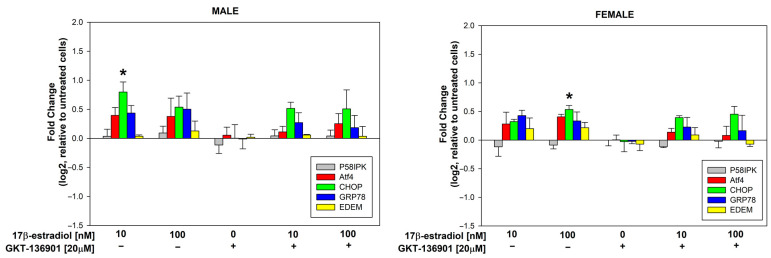
log2 fold change representing the mRNA expression of genes related to ER stress/UPR initiation in cells derived from male and female porcine thyroid. Cells were incubated with 17β-estradiol at concentrations of 0.0 nM, 10 nM, or 100 nM, with or without GKT-136901 (20 µM). mRNA expression was calculated relative to the untreated control cells. Bars represent the mean ± SE of three independent experiments. * *p* < 0.05 vs. respective control (without 17β-estradiol).

**Figure 8:**
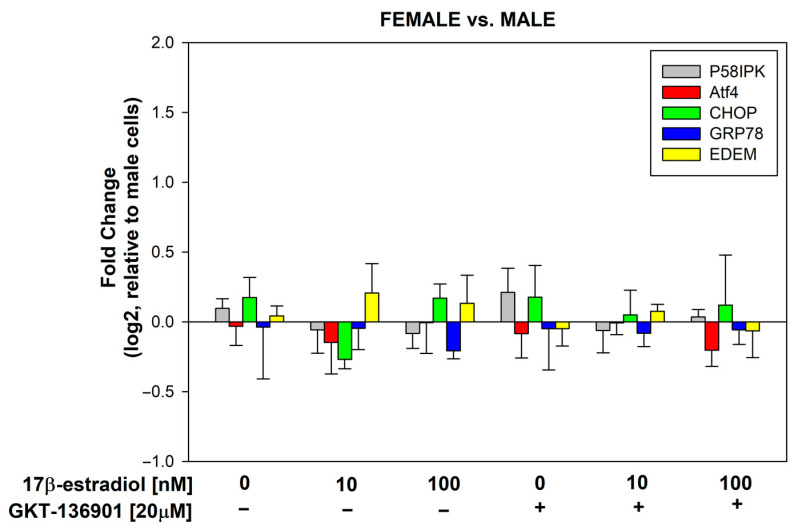
log2 fold change representing the mRNA expression of genes related to ER stress/UPR initiation in female porcine thyroid cells relative to male porcine thyroid cells. Cells were incubated with 17β-estradiol at concentrations of 0.0 nM, 10 nM, or 100 nM, with or without GKT-136901 (20 µM). Bars represent the mean ± SE of three independent experiments.

17β-estradiol concentrations of 10 and 100 nmol/L were added to the male and female porcine thyroid primary cell lines for 48 h to assess its effect on the expression of genes involved in the cellular response to stress within the ER, i.e., P58IPK, Atf4, CHOP, GRP78, and EDEM. Additionally, to evaluate the potential involvement of NOX4 in the processes of ER stress, GKT-136901 (selective NOX4 inhibitor) was used in the concentration of 20 µM. The analysis of the RT-PCR results showed that 17β-estradiol in the concentration of 10 nM causes a statistically significant increase in the expression of the CHOP gene in male cells. In female cells, a statistically significant increase in the expression of the CHOP gene was caused by 17β-estradiol in a concentration of 100 nM (Figure 7). The use of GKT-136901 did not change the level of thyroid-specific gene expression (Figure 8).

## 4. Discussion

As mentioned in the Introduction, women are notably more susceptible to thyroid disorders than men, and this difference relates to both thyroid tumors and autoimmune diseases [26,27]. This disparity in prevalence has been partially attributed to hormonal differences, as estrogens have been shown to stimulate the proliferation of thyroid cells [28]. The interaction between estrogen and thyroid function has been a subject of intensive research, with numerous studies demonstrating the influence of estrogens, particularly 17β-estradiol, on the pathogenesis of thyroid diseases [10,11,12]. The role of 17β-estradiol in promoting thyroid cancer development is particularly notable, with evidence suggesting that it affects tumor growth through receptor-mediated pathways, activating key genes involved in cell proliferation [13,29,30]. This correlation is supported by findings that elevated estradiol levels are associated with increased thyroid hormone concentrations, suggesting that estrogen may modulate thyroid function through multiple pathways, including its effects on TSH and TPO [31,32]. Furthermore, estradiol influence extends to the tumor microenvironment, where it promotes conditions favorable for thyroid cancer progression, including inflammation, hypoxia, and angiogenesis [33].

Recent studies further emphasize the complex relationship between estrogen and thyroid diseases, particularly autoimmune conditions such as Hashimoto’s thyroiditis. In cases of polycystic ovary syndrome (PCOS), where hormonal imbalances are common, women with higher estradiol levels are more likely to exhibit positive anti-TPO antibodies, indicating a potential link between estrogen and autoimmune thyroid disease [34].

However, the mechanisms through which estrogen exacerbates these processes remain unclear, with studies suggesting that reactive oxygen species (ROS) generated by estrogen action may contribute to the observed sexual dimorphism in thyroid disease prevalence [15,16,17,18].

In the present work, we observed that 17β-estradiol induces a dose-dependent increase in the expression of NOX4 and DUOX1 in thyroid follicular cells from both males and females. The upregulation of NOX/DUOX expression was accompanied by a substantial rise in ROS levels, with a two-fold increase observed at 10 nM and a three-fold increase at 100 nM of 17β-estradiol. We observed that, under physiological conditions, ROS levels are independent of NOX4 activity; however, treatment with the NOX4 inhibitor GKT-136901 significantly reduced ROS levels in both male and female thyroid cells incubated with 10 and 100 nM of 17β-estradiol. These results suggest that hyperestrogenism induces oxidative stress in the thyroid, at least partially, through NOX4 stimulation, contributing to a redox imbalance. Our findings indicate that this mechanism of NOX4-mediated ROS generation does not significantly differ between male and female thyroid cells, implying that the influence of elevated estrogen on oxidative stress is consistent across sexes. These observations underscore the role of NOX4 in estrogen-induced oxidative stress, which may be particularly relevant in conditions characterized by elevated estrogen levels, such as pregnancy or hormone therapy. Such an observation is particularly important given the overexpression of NOX4 in thyroid cancer, especially in papillary thyroid carcinomas (PTC), where NOX4 has been linked to cancer pathogenesis through its role in generating H_2_O_2_ and promoting oxidative stress [23]. The correlation between NOX4 overexpression and the BRAFV600E mutation in PTC further underscores the significance of NOX4 in tumor development and aggressiveness [35].

Importantly, in contrast to our previous findings in rat thyroid cells [16], we did not observe statistically significant differences in the upregulation of NOX/DUOX expression by exogenous 17β-estradiol between male and female cells. The male thyroids used in our study were collected from animals that, according to general recommendations, are routinely castrated at 3–7 days of age. As a result, these male thyroids (in contrast to non-castrated animals) were exposed to a different hormonal state during adult life, characterized by a relative predominance of estrogen compared to testosterone. It is important to note that in our previous study with rat thyroids, the animals were not castrated; thus, the observed differences in NOX/DUOX expression between females and males likely reflected natural hormonal differences. This hormonal distinction may explain why we did not observe as pronounced sex-related differences in the current study as we did previously. Moreover, porcine and rat thyroid cells can exhibit variations in estrogen receptor sensitivity, gene regulation, and cellular signaling pathways. These differences can affect how thyroid cells from various species’ respond to hormonal stimuli such as 17β-estradiol. The estrogen receptors (α and β) in porcine cells may have different activity levels or expression patterns compared to rat cells, leading to a reduced or altered response in NOX/DUOX regulation, which could explain the absence of sex-specific effects in our study.

In our present work, we confirmed a strong stimulatory effect of 17β-estradiol on thyroid cells, as demonstrated by the increased expression of NKX2-1, Tg, and especially the statistically significant increased expression of TPO in both male and female cells. NKX2-1 (also known as TTF-1) is a critical transcription factor that regulates the expression of thyroid-specific genes, including Tg and TPO, which are essential for thyroid hormone synthesis. Tg serves as a precursor for the synthesis of thyroid hormones, i.e., triiodothyronine (T3) and thyroxine (T4), while TPO catalyzes the iodination of Tg, a crucial step in thyroid hormone biosynthesis. The upregulation of these genes suggests that estradiol enhances the thyroid hormone synthesis process. Our results align with findings from studies in a mouse model of early pregnancy exposed to elevated 17β-estradiol [32]. In that model, enhanced expression of TPO, PAX8, and significantly increased T4 and free thyroxine (FT4) levels were observed in the thyroids of offspring [31]. In another research conducted on female rats, 17β-estradiol increased concentrations of both T3 and T4 [32]. These findings are consistent with our results, reinforcing the role of estradiol in regulating key genes involved in thyroid hormone production and suggesting a potential epigenetic mechanism underlying this regulation.

The results of our present research indicate that while 17β-estradiol stimulates the expression of key thyroid-specific genes such as TPO, no significant stimulation of NIS expression was observed. This finding aligns with previous studies suggesting that high concentrations of 17β-estradiol can inhibit NIS expression through the estrogen receptor β-mediated pathway, potentially leading to thyroid hormone fluctuations during pregnancy [36]. Given that NIS is crucial for iodide uptake in thyroid hormone synthesis, the suppression of its expression by estradiol could explain the observed hormonal imbalances in high-estrogen states, such as pregnancy, further emphasizing the complex role of estradiol in regulating thyroid function.

In our research, we have also assessed the effect of 17β-estradiol on the expression of genes involved in the cellular response to stress within the ER, specifically P58IPK, Atf4, CHOP, GRP78, and EDEM. These genes are integral to the UPR, a key cellular mechanism activated to maintain ER homeostasis when there is stress related to protein synthesis, folding, and quality control [37,38]. Disruptions in this process can lead to ER stress, which may impair the normal functioning of thyrocytes, the cells specialized for thyroid hormone synthesis/secretion.

Our findings demonstrate that 17β-estradiol at a concentration of 10 nM caused a statistically significant increase in the expression of the CHOP gene in male cells, whereas a significant increase in CHOP expression was observed in female cells only at a higher concentration of 100 nM. CHOP (CAAT/enhancer binding protein (C/EBP) homologous protein) is a pro-apoptotic transcription factor that plays a critical role in the UPR [39]. Its upregulation indicates severe or prolonged ER stress, leading to apoptosis when the cell is unable to restore homeostasis. The differential sensitivity to 17β-estradiol between male and female cells in our study may suggest sex-specific regulatory mechanisms in the ER stress response, potentially mediated by estrogen receptors and downstream signaling pathways.

Additionally, we studied P58IPK, which acts as a molecular chaperone that inhibits ER stress-induced apoptosis, promoting cell survival during UPR activation. Atf4 is another critical component of the UPR that helps restore protein homeostasis by activating genes involved in amino acid metabolism and redox balance. GRP78 (BiP) is a chaperone protein that binds to misfolded proteins and initiates the UPR, while EDEM is involved in ER-associated degradation (ERAD), targeting misfolded glycoproteins for degradation. Though these genes are essential for mitigating ER stress, we did not observe significant changes in their expression under the tested conditions, suggesting that the stress induced by 17β-estradiol in thyroid cells may be more specifically linked to apoptosis via the CHOP pathway.

The role of NOX4 in contributing to ER stress is well documented, as its production of H_2_O_2_ can act as a signaling molecule to initiate the UPR and autophagy [40,41]. The increased expression of CHOP in our study could potentially be linked to increased ROS production, which exacerbates ER stress. This relationship is particularly important when considering the link between ER stress and thyroid autoimmunity, as ER stress has been implicated in the pathogenesis of autoimmune thyroid diseases [42,43]. The sex-specific differences observed in the present study in the response to 17β-estradiol may provide further insight into the higher prevalence of thyroid diseases in females, highlighting the potential role of estrogen in modulating the ER stress response in thyroid cells.

Our study has some clear limitations. The first one is that this is an in vitro study with primary cell lines. The second relates to the fact that, while we used a species (porcine) highly similar to humans in terms of thyroid volume, hormone synthesis, and other characteristics [44], the hormonal state of male and female pigs may not fully reflect the hormonal differences between adult women and men. Additionally, as mentioned earlier, the male pigs were castrated, which may have further altered the hormonal profile to which the tissue is exposed, potentially affecting the outcomes of our research.

To sum up, 17β-estradiol significantly upregulates key thyroid-specific genes, particularly TPO, and has a major impact on the redox state of thyroid cells through the stimulation of NOX/DUOX expression. Additionally, 17β-estradiol influences the expression of genes involved in the cellular response to ER stress, especially CHOP.

In conclusion, estrogen excess may contribute to thyroid disease development via such possible mechanisms as the upregulation of key thyroid-specific genes, particularly TPO, and of genes involved in the cellular response to ER stress, especially CHOP, as well as by the stimulation of NOX/DUOX system with consequent ROS overproduction. At the same time, these mechanisms may play a certain role in the higher prevalence of thyroid diseases, such as thyroid cancer and autoimmune thyroid diseases, in women as compared to men.

## Data Availability

The original contributions presented in the study are included in the article, further inquiries can be directed to the corresponding author.
